# Anxiety, irritability, and agitation as indicators of bipolar mania with depressive symptoms: a post hoc analysis of two clinical trials

**DOI:** 10.1186/s40345-017-0103-7

**Published:** 2017-11-06

**Authors:** Trisha Suppes, Jonas Eberhard, Ole Lemming, Allan H. Young, Roger S. McIntyre

**Affiliations:** 1 VA Palo Alto Health Care System and Department of Psychiatry and Behavioral Sciences, Stanford University School of Medicine, Stanford, CA USA; 2 H. Lundbeck A/S, Valby, Copenhagen, Denmark; 3 Department of Clinical Sciences, Lund University, Lund, Sweden; 4 Department of Psychological Medicine, Institute of Psychiatry, Psychology & Neuroscience (IoPPN), King’s College London, London, UK; 5 Mood Disorders Psychopharmacology Unit, University Health Network, University of Toronto, Toronto, ON Canada

**Keywords:** Bipolar disorder, Mixed features, Depression, Anxiety, Irritability, Agitation, Remission

## Abstract

**Background:**

Symptoms of anxiety, irritability, and agitation (AIA) are prevalent among patients with bipolar I disorder (BD-I) mania with depressive symptoms, and could potentially be used to aid physicians in the identification of this more severe form of BD-I. Using data from two clinical trials, the aims of this post hoc analysis were to describe the phenomenology of bipolar mania in terms of AIA and depressive symptoms, and to evaluate the influence of these symptoms on the likelihood of remission during treatment.

**Methods:**

Patients with a BD-I manic or mixed episode (Diagnostic and Statistical Manual of Mental Disorders IV criteria) were randomised to 3 weeks of double-blind treatment with asenapine, placebo, or olanzapine (active comparator). Anxiety was defined as a score of ≥3 on the Positive and Negative Syndrome Scale ‘anxiety’ item, irritability as a score of ≥4 on the Young Mania Rating Scale (YMRS) ‘irritability’ item, and agitation as a score of ≥3 on the YMRS ‘increased motor activity–energy’ item. Depressive symptoms were defined as a score of ≥1 on three or more individual Montgomery–Åsberg Depression Rating Scale (MADRS) items, or a MADRS Total score of ≥20.

**Results:**

A total of 960 patients with BD-I were analysed, 665 with a manic episode and 295 with a mixed episode. At baseline, 61.4% had anxiety, 62.4% had irritability, 76.4% had agitation, and 34.0% had all three AIA symptoms (‘severe AIA’); 47.3% had three or more depressive symptoms, and 13.5% had a MADRS total score of ≥20. Anxiety, irritability, and severe AIA (but not agitation) were statistically significantly more common in patients with depressive symptoms. Patients with anxiety or severe AIA at baseline were statistically significantly less likely to achieve remission (YMRS total <12). In general, remission rates were higher with asenapine and olanzapine than with placebo, irrespective of baseline AIA or depressive symptoms.

**Conclusions:**

Assessment of AIA symptoms in bipolar mania could enable physicians to identify patients with more severe depressive symptoms, allowing for appropriate intervention. Assessment and monitoring of AIA may help physicians to predict which patients may be harder to treat and at risk for self-harm.

*Trial registration* ClinicalTrials.gov NCT00159744, NCT00159796. Registered 8 September 2005 (retrospectively registered)

## Background

Bipolar I disorder (BD-I) is an illness that comprises more than just two discrete poles; between the extremes of ‘pure’ mania and ‘pure’ depression, patients can experience ‘mixed’ states in which various manic and depressive symptoms are present at the same time. Mixed states were described by Kraepelin in the early 20th century, and have been well characterised and operationally defined in the 100 years since (Kraepelin 1921; Swann et al. 2013). The fourth edition of the Diagnostic and Statistical Manual of Mental Disorders (DSM-IV) defined a ‘mixed episode’ as the coexistence of the full symptomatology of a manic and a depressive episode—a restrictive, categorical approach that neglects those patients with subsyndromal depressive symptoms (Vieta and Valentí 2013; American Psychiatric Association 1994). Since the DSM-IV, studies have shown that this definition does not reflect clinical reality and that patients with bipolar disorder [as well as patients with major depressive disorder (MDD)] have a complex symptom presentation (Suppes et al. 2005; Vieta and Valentí 2013; Targum and Nierenberg 2011). Consequently, the fifth edition of the DSM (DSM-5) changed to a dimensional approach for the diagnosis of BD-I (Vieta and Valentí 2013). The DSM-5 includes a ‘with mixed features’ specifier that applies to a manic or hypomanic episode with three or more depressive symptoms (or, alternatively, to a depressive episode with three or more manic symptoms) (American Psychiatric Association 2013). In a retrospective chart review, three times as many patients with bipolar disorder were diagnosed with mixed features using the DSM-5 criteria compared with the DSM-IV text revision (DSM-IV-TR) criteria for a mixed episode (Shim et al. 2015). In an observational study, around half of patients with DSM-IV bipolar mania did not meet the criteria for DSM-5 bipolar mania, due to the additional requirement of increased activity or energy levels (Machado-Vieira et al. 2017). However, 45% of these patients now met the DSM-5 criteria for a major depressive episode with mixed features, showing the value of the mixed features specifier for making diagnoses across the continuum of mood disorders (Machado-Vieira et al. 2017).

Patients with BD-I mixed states have a worse clinical course than those with ‘pure’ manic states, since they tend to experience more episodes, and since episodes are of longer duration (Swann et al. 2013). Mania with depressive symptoms is also associated with a high rate of comorbid conditions, such as anxiety disorders and substance abuse (Swann et al. 2013). These patients are harder to treat and physicians of patients meeting the DSM-5 criteria for mania ‘with mixed features’ report greater treatment dissatisfaction than do physicians of patients without mixed features (Swann et al. 2013; Young and Eberhard 2015). Furthermore, there is a higher risk of suicidality among patients with mixed features; these patients are more likely to have made at least one suicide attempt during their lifetime, and during their current manic episode, compared with patients without mixed features (Swann et al. 2013; Young and Eberhard 2015). Thus, the presence of depressive symptoms during a manic episode is indicative of a more severe form of BD-I.

The symptoms observed in manic episodes and depressive episodes are not mutually exclusive, with anxiety, irritability, and agitation (AIA), for example, occurring in both mania and depression. Since they are not specific to either pole, AIA symptoms are not included in the DSM-5 ‘with mixed features’ specifier (Cassidy 2010; Koukopoulos et al. 2013). [Anxiety is, however, covered by the DSM-5 ‘with anxious distress’ specifier (American Psychiatric Association 2013)]. According to physicians, AIA symptoms show an increased prevalence, and greater severity, in patients with mixed features in BD-I mania compared with those without mixed features (Young and Eberhard 2015). Similarly, in an online survey, patients with mania with depressive symptoms self-reported the combination of ‘anxiety or worry’ and ‘irritability or agitation’ more frequently than those without depressive symptoms (Vieta et al. 2014). A recent prospective study from the Stanley Bipolar Network found increased irritability in bipolar patients with mixed depression compared with pure depression (Miller et al. 2016). Due to their high frequency among such patients, AIA symptoms could potentially be used as indicators (albeit non-specific indicators) to aid physicians in the identification of patients whose mania is complicated by depressive symptoms.

Using data from two pivotal, randomised, placebo-controlled asenapine trials, the aims of the present post hoc analysis were (1) to describe the phenomenology of bipolar mania in terms of AIA and depressive symptoms, (2) to evaluate if the presence of AIA and depressive symptoms can be used to predict non-remission, and (3) to evaluate the effect of asenapine, placebo, and olanzapine on remission in patients with AIA, and in patients with depressive symptoms.

## Methods

This was a post hoc analysis of patient-level data from two 3-week, randomised, placebo-controlled trials (ARES 7501004 and ARES 7501005; ClinicalTrials.gov identifiers NCT00159744 and NCT00159796, respectively) of asenapine in patients with BD-I experiencing an acute DSM-IV manic or mixed episode (McIntyre et al. 2009, 2010). Olanzapine was included as active comparator.

### Study design and patients

The two primary studies had identical designs, which have been described previously (McIntyre et al. 2009, 2010). In brief, eligible patients were aged ≥18 years with a current DSM-IV diagnosis of BD-I. Patients were required to have a manic or mixed episode that began in the 3 months prior to screening, a Young Mania Rating Scale (YMRS; Young et al. 1978). Total score of ≥20 at screening and baseline, and a documented history of at least one previous moderate-to-severe mood episode, with or without psychotic features. Patients were excluded if they had a primary diagnosis other than BD-I, a psychotic disorder, rapid-cycling bipolar disorder, or substance abuse or dependence.

Eligible patients entered a single-blind placebo run-in (washout) period of up to 7 days in an inpatient treatment centre. Following the run-in period, patients were randomised in a 2:1:2 ratio to 3 weeks of double-blind treatment with oral asenapine (20 mg on Day 1, flexible-dose 10 or 20 mg/day thereafter; dose divided across morning and evening), placebo, or olanzapine (15 mg on Day 1, flexible-dose 5–20 mg thereafter; once daily). Patients remained in the inpatient centre for at least the first 7 days of treatment, after which they could complete the trial as outpatients if they were deemed stable and suitable for discharge by the investigator. Benzodiazepines (lorazepam ≤4 mg/day for agitation; temazepam ≤30 mg/day for insomnia; or others with comparable half-lives) were permitted for the first 7 days of treatment only.

### Assessments

In the two primary studies, efficacy was assessed using psychiatric rating scales including the YMRS, the Clinical Global Impressions-Bipolar version (CGI-BP; Spearing et al. 1997; Guy 1976), the Montgomery–Åsberg Depression Rating Scale (MADRS; Montgomery and Åsberg 1979), and the Positive and Negative Syndrome Scale (PANSS; Kay et al. 1987). YMRS and CGI-BP assessments were made at screening, baseline, and Days 2, 4, 7, 14, and 21 (or end of treatment). MADRS assessments were made at baseline, Day 7, and at Day 21 or study endpoint.

In this post hoc analysis, the presence or absence of AIA symptoms at baseline was assessed using the following definitions. Anxiety was defined as a score of ≥3 (“mild”) on the PANSS anxiety item (item G2; possible score range from 1 “absent” to 7 “extreme”; Kay et al. 1987). Irritability was defined as a score of ≥4 (“irritable at times during interview, recent episodes of anger or annoyance on ward”) on the YMRS irritability item (item 5; possible score range from 0 “absent” to 8 “hostile, uncooperative; interview impossible”; Young et al. 1978). Agitation was defined as a score of ≥3 (“excessive energy, hyperactive at times, restless [can be calmed]”) on the YMRS increased motor activity–energy item (item 2; possible score range from 0 “absent” to 4 “motor excitement; continuous hyperactivity [cannot be calmed]”; Young et al. 1978). ‘Severe AIA’ was defined as meeting the above criteria for all three AIA symptoms.

Two definitions were used, in separate analyses, for the presence of depressive symptoms at baseline: (1) a score of ≥1 on three or more individual MADRS items; (2) a MADRS Total score of ≥20.

Two definitions were used, in separate analyses, for YMRS remission: (1) a YMRS Total score of <12 at endpoint (Day 21); (2) a YMRS Total score of <8 at endpoint (Day 21).

Safety and tolerability were assessed in the primary studies; please refer to the primary sources for these analyses (McIntyre et al. 2009, 2010).

### Statistical analyses

This post hoc analysis was performed on the full analysis set (FAS), which comprised all randomised patients who took at least one dose of study medication, and had at least one valid post-baseline YMRS assessment.

For baseline analyses of the incidence of AIA symptoms stratified by the number and severity of depressive symptoms, between-group comparisons were made using a Chi squared test. Incidence of remission, stratified by the presence or absence of baseline AIA and depressive symptoms, was analysed using two criteria—YMRS Total score of <12, and YMRS Total score of <8, at Day 21—also using a Chi squared test for between-group comparisons. Due to repeated testing, a significance level of 0.01 was applied in the remission analyses (Bonferroni adjustment for multiplicity); a significance level of 0.05 was applied elsewhere. A footnote on relevant comparisons indicates where the significance level is 0.01.

The AIA parameters that were found to be statistically significant predictors of non-remission were then examined using an analysis of covariance (ANCOVA), in order to determine the effects of treatment (asenapine, placebo, or olanzapine) on change in YMRS Total score over time. Between-group comparisons were made using a *t* test.

## Results

### Disposition

Across the two primary studies, a total of 977 patients were randomised and 976 were treated. The FAS comprised 960 patients (480 from each study), split into asenapine (*n* = 372), placebo (*n* = 197), and olanzapine (*n* = 391) treatment groups.

#### Baseline characteristics: phenomenology of bipolar mania in terms of AIA and depressive symptoms

Patient baseline demographic and clinical characteristics are presented in Table 1. Slightly over half of patients were male, and the majority were unemployed. The ratio of manic to mixed episodes of BD-I was approximately 7:3. On average, patients had pronounced mania at baseline (mean [SD] YMRS Total score of 28.9 [6.2] and CGI-BP Mania severity of 4.6 [0.8]—markedly ill), and mild depression (mean [SD] MADRS Total score of 11.3 [7.3] and CGI-BP Depression severity of 2.1 [1.3]—minimally ill). Patients were well matched between treatment groups for all measurements.

**Table 1:** Baseline demographic and clinical characteristics

	Asenapine (*n* = 372)	Placebo (*n* = 197)	Olanzapine (*n* = 391)	Total (*N* = 960)
Male, *n* (%)	203 (54.6)	98 (49.7)	230 (58.8)	531 (55.3)
Age (years), median (range)	40 (18–76)	39 (18–69)	39 (18–67)	39 (18–76)
Unemployed, *n* (%)	270 (72.6)	139 (70.6)	285 (72.9)	694 (72.3)
DSM-IV BD-I diagnosis, *n* (%)				
Manic episode	265 (71.2)	131 (66.5)	269 (68.8)	665 (69.3)
Mixed episode	107 (28.8)	66 (33.5)	122 (31.2)	295 (30.7)
YMRS total score^a^, mean (SD)	28.8 (6.2)	28.7 (6.2)	29.2 (6.3)	28.9 (6.2)
MADRS total score^a^, mean (SD)	10.8 (7.0)	12.4 (7.8)	11.3 (7.3)	11.3 (7.3)
CGI-BP mania severity score^a^, mean (SD)	4.7 (0.8)	4.6 (0.8)	4.6 (0.8)	4.6 (0.8)
CGI-BP depression severity score^a^, mean (SD)	2.0 (1.2)	2.2 (1.3)	2.1 (1.3)	2.1 (1.3)
Number of depressive symptoms^b^, *n* (%)				
0–2	201 (54.0)	100 (50.8)	205 (52.4)	506 (52.7)
3 or more	171 (46.0)	97 (49.2)	186 (47.6)	454 (47.3)
Severity of depressive symptoms, n (%)				
MADRS Total score <20	328 (88.2)	164 (83.2)	338 (86.4)	830 (86.5)
MADRS Total score ≥20	44 (11.8)	33 (16.8)	53 (13.6)	130 (13.5)
Anxiety^c^, *n* (%)				
Absent or minimal	144 (38.7)	73 (37.1)	150 (38.4)	367 (38.2)
Present	227 (61.0)	124 (62.9)	238 (60.9)	589 (61.4)
Not rated	1 (0.3)	0 (0.0)	3 (0.8)	4 (0.4)
Irritability^d^, *n* (%)				
Absent or low	150 (40.3)	65 (33.0)	134 (34.3)	349 (36.4)
Present	218 (58.6)	128 (65.0)	253 (64.7)	599 (62.4)
Not rated	4 (1.1)	4 (2.0)	4 (1.0)	12 (1.3)
Agitation^e^, *n* (%)				
Absent or low	83 (22.3)	48 (24.4)	84 (21.5)	215 (22.4)
Present	285 (76.6)	145 (73.6)	303 (77.5)	733 (76.4)
Not rated	4 (1.1)	4 (2.0)	4 (1.0)	12 (1.3)
Severe AIA^f^, *n* (%)				
Not present	247 (66.4)	123 (62.4)	248 (63.4)	618 (64.4)
Present	120 (32.3)	70 (35.5)	136 (34.8)	326 (34.0)
One or more symptom not rated	5 (1.3)	4 (2.0)	7 (1.8)	16 (1.7)

AIA, anxiety, irritability, and agitation; BD-I, bipolar I disorder; CGI-BP, Clinical Global Impressions-Bipolar version; DSM-IV, Diagnostic and Statistical Manual of Mental Disorders, Fourth Edition; MADRS, Montgomery–Åsberg Depression Rating Scale; PANSS, Positive and Negative Syndrome Scale; SD, standard deviation; YMRS, Young Mania Rating Scale

^a^For patients missing a value at baseline, the value was used from the screening visit

^b^Based on the number of individual MADRS items with a score of ≥1

^c^Based on the PANSS anxiety item: ‘absent or minimal’ indicates a score of ≤2; ‘present’ indicates a score of ≥3

^d^Based on the YMRS irritability item: ‘absent or low’ indicates a score of ≤3; ‘present’ indicates a score of ≥4

^e^Based on the YMRS increased motor activity–energy item: ‘absent or low’ indicates a score of ≤2; ‘present’ indicates a score of ≥3

^f^Meeting the above criteria for all three AIA symptoms

**Fig. 1:**
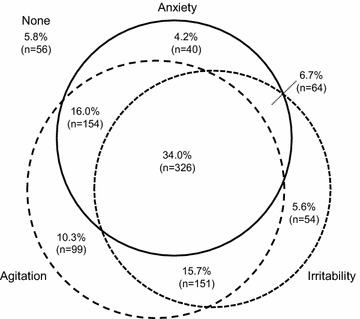
Venn diagram displaying the overlap of comorbid AIA symptoms in BD-I mania. Sixteen patients (1.7%) were missing a value for one or more AIA symptom at baseline and are not shown on the figure. Anxiety was defined as a PANSS anxiety item score of ≥3 at baseline. Irritability was defined as a YMRS irritability item score of ≥4 at baseline. Agitation was defined as a YMRS increased motor activity–energy item score of ≥3 at baseline. Percentages are calculated from the full analysis set, which comprised 960 patients. AIA, anxiety, irritability, and agitation; BD-I, bipolar I disorder; PANSS, Positive and Negative Syndrome Scale; YMRS, Young Mania Rating Scale

Overall, 589 patients (61.4%) had anxiety at baseline, 599 patients (62.4%) had irritability at baseline, and 733 patients (76.4%) had agitation at baseline. Severe AIA (i.e., all three AIA symptoms) was present in 326 patients (34.0%). The proportion of patients with each AIA symptom, and the overlap between symptoms, is shown in Fig. 1.

Considering depressive symptoms, 454 patients (47.3%) had three or more depressive symptoms at baseline based on individual MADRS items, and 130 patients (13.5%) had a MADRS Total score of ≥20.

**Fig. 2:**
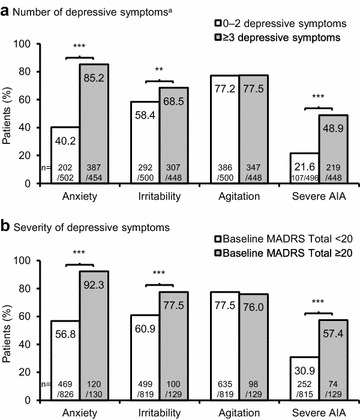
Incidence of baseline AIA symptoms in BD-I mania, stratified by the number/severity of depressive symptoms. **p < 0.01, ***p < 0.001, Chi squared test. Anxiety was defined as a PANSS anxiety item score of ≥3 at baseline. Irritability was defined as a YMRS irritability item score of ≥4 at baseline. Agitation was defined as a YMRS increased motor activity–energy item score of ≥3 at baseline. Severe AIA was defined as meeting the above criteria for all three AIA symptoms. Patients missing a value for the corresponding symptom at baseline were not included in the analysis. ^a^ Based on the number of individual MADRS items with a score of ≥1. AIA, anxiety, irritability, and agitation; BD-I, bipolar I disorder; MADRS, Montgomery–Åsberg Depression Rating Scale; PANSS, Positive and Negative Syndrome Scale; YMRS, Young Mania Rating Scale. **a** Number of depressive symptoms of 0-2 symptoms versus 3 or more symptoms, and **b** severity of depressive symptoms baseline MADRS <20 versus ≥ to 20

Figure 2 shows the relationships between depressive symptoms and AIA symptoms at baseline. Anxiety and irritability were statistically significantly more common in patients with depressive symptoms at baseline, both for patients with three or more individual depressive symptoms, and for patients with a MADRS Total score of ≥20. Agitation, in contrast, was not more common in patients with depressive symptoms at baseline. Finally, severe AIA (all three symptoms) was statistically significantly more common in patients with depressive symptoms at baseline.

#### Can the presence of AIA and depressive symptoms be used to predict non-remission?

**Fig. 3:**
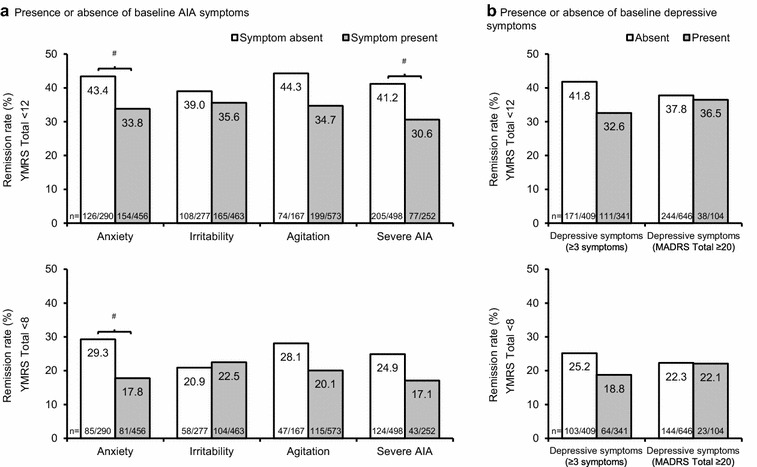
Incidence of remission in BD-I mania, stratified by baseline AIA/depressive symptoms. ^#^ *p*-value significant (when adjusted for multiplicity), Chi squared test. Anxiety was defined as a PANSS anxiety item score of ≥3 at baseline. Irritability was defined as a YMRS irritability item score of ≥4 at baseline. Agitation was defined as a YMRS increased motor activity–energy item score of ≥3 at baseline. Severe AIA was defined as meeting the above criteria for all three AIA symptoms. Patients missing a value for the corresponding symptom at baseline were not included in the analysis. Two definitions were used for remission: a YMRS Total score of <12 at Day 21 (upper figures); and a YMRS Total score of <8 at Day 21 (lower figures) (observed cases). Depressive symptoms were defined in two ways: ≥3 individual MADRS items with a score of ≥1, or a MADRS Total score of ≥20 at baseline. AIA, anxiety, irritability, and agitation; BD-I, bipolar I disorder; MADRS, Montgomery–Åsberg Depression Rating Scale; PANSS, Positive and Negative Syndrome Scale; YMRS, Young Mania Rating Scale. **a** Presence or absence of baseline AIA symptoms and **b** presence or absence of baseline depressive symptoms

As shown in Fig. 3a, patients with anxiety at baseline were statistically significantly less likely to achieve remission than those without anxiety at baseline, according to both definitions of remission (YMRS Total score of <12 and <8 at Day 21). The presence of severe AIA at baseline also had a statistically significant negative effect on remission, when defined as a YMRS Total score of <12. The presence of agitation at baseline reduced the incidence of remission by 8–10% points, but this did not meet the criteria for statistical significance. The only AIA symptom without a notable effect on remission was irritability.

As shown in Fig. 3b, the presence of three or more depressive symptoms at baseline reduced the incidence of remission by 6–9% points, but this did not meet the criteria for statistical significance. In contrast, the presence of depressive symptoms defined by a MADRS Total score of ≥20 had no notable effect on remission rate.

#### Effect of treatment on remission in patients with AIA and depressive symptoms

**Fig. 4:**
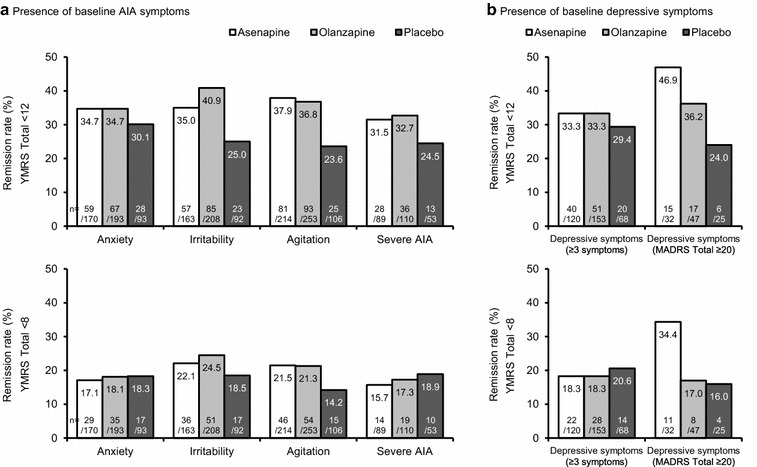
Incidence of remission by treatment group in BD-I mania, stratified by baseline AIA/depressive symptoms. Anxiety was defined as a PANSS anxiety item score of ≥3 at baseline. Irritability was defined as a YMRS irritability item score of ≥4 at baseline. Agitation was defined as a YMRS increased motor activity–energy item score of ≥3 at baseline. Severe AIA was defined as meeting the above criteria for all three AIA symptoms. Patients missing a value for the corresponding symptom at baseline were not included in the analysis. Two definitions were used for remission: a YMRS Total score of <12 at Day 21 (upper figures); and a YMRS Total score of <8 at Day 21 (lower figures) (observed cases). Depressive symptoms were defined in two ways: ≥3 individual MADRS items with a score of ≥1, or a MADRS Total score of ≥20 at baseline. AIA, anxiety, irritability, and agi- tation; BD-I, bipolar I disorder; MADRS, Montgomery–Åsberg Depression Rating Scale; PANSS, Positive and Negative Syndrome Scale; YMRS, Young Mania Rating Scale. **a** Presence of baseline AIA symptoms and **b** presence of baseline depressive symptoms

With remission defined as a YMRS Total score of <12 at Day 21, remission rates were higher with asenapine and olanzapine than with placebo among patients with anxiety, irritability, agitation, or severe AIA at baseline, as shown in Fig. 4a. With remission defined as a YMRS Total score of <8 at Day 21, remission rates were only higher than placebo with asenapine and olanzapine among patients with irritability or agitation at baseline. For patients with depressive symptoms at baseline, remission rates were generally higher with asenapine and olanzapine than with placebo, as shown in Fig. 4b. These differences were not tested for statistical significance.

**Fig. 5:**
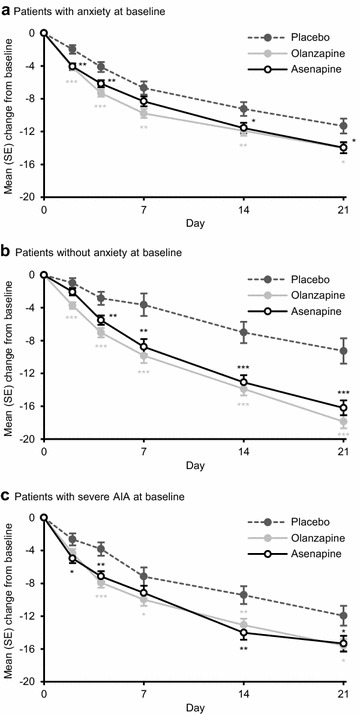
Change from baseline in YMRS Total score by treatment group in BD-I mania. *p < 0.05, **p < 0.01, ***p < 0.001 versus placebo, *t* test. Anxiety was defined as a PANSS anxiety item score of ≥3 at baseline. Irritability was defined as a YMRS irritability item score of ≥4 at baseline. Agitation was defined as a YMRS increased motor activity–energy item score of ≥3 at baseline. Severe AIA was defined as meeting the above criteria for all three AIA symptoms. Patients missing a value for the corresponding symptom at baseline were not included in the analysis. AIA, anxiety, irritability, and agitation; BD-I, bipolar I disorder; PANSS, Positive and Negative Syndrome Scale; SE, standard error; YMRS, Young Mania Rating Scale. **a** Patients with anxiety at baseline, **b** patients without anxiety at baseline, and **c** patients with severe AIA at baseline

Since baseline anxiety and severe AIA were identified as statistically significant predictors of non-remission (see Fig. 3a), stratified analyses according to feature present or not present were carried out, to determine the effect of treatment on change in YMRS Total score over time. As shown in Fig. 5, asenapine and olanzapine produced a statistically significant benefit on YMRS Total score at the majority of time points among patients with anxiety at baseline (Day 21 effect sizes: 0.33 for asenapine, and 0.34 for olanzapine). The benefits were even greater among patients without anxiety at baseline (Day 21 effect sizes: 0.76 for asenapine, and 0.95 for olanzapine). A statistically significant benefit on YMRS Total score was also observed at the majority of time points among patients with severe AIA at baseline (Day 21 effect sizes: 0.42 for asenapine, and 0.45 for olanzapine).

## Discussion

This post hoc analysis of two randomised, controlled trials revealed that symptoms of anxiety, irritability, and agitation were present in around two-thirds-to-three-quarters of patients with BD-I mania. In addition, around a third of patients had all three AIA symptoms. While different studies have defined the presence of AIA symptoms in different ways, there is agreement in the literature that AIA symptoms are common among patients with BD-I mania (Hantouche et al. 2006; Vieta et al. 2014; Young and Eberhard 2015).

The present analysis also showed that half of patients with BD-I mania had at least three depressive symptoms, whereas less than one in seven patients had a MADRS Total score of ≥20. A previous post hoc analysis in the same dataset found that 4–34% of patients met proxy criteria for the DSM-5 ‘with mixed features’ specifier, depending on the severity cut-offs used (McIntyre et al. 2013a). In general, the incidence of depressive symptoms among bipolar disorder patients with mania is around 30–40%, though the incidence varies widely according to how the presence of depressive symptoms is defined (McElroy et al. 1992; Akiskal et al. 1998; Hantouche et al. 2006; Azorin et al. 2009; Vieta et al. 2014; Young and Eberhard 2015; McIntyre et al. 2015). In a prospective study of patients with bipolar hypomania, the likelihood of having depressive symptoms was significantly greater for women (72%) than for men (42%) (Suppes et al. 2005).

With regard to the relationship between AIA and depressive symptoms, the analysis showed that anxiety, irritability, and the AIA combination—but not agitation alone—were more common in patients with depressive symptoms at baseline. This replicates the findings of a naturalistic study, in which anxiety and irritability were more common in BD-I patients with three or more depressive symptoms, whereas agitation was not (Young and Eberhard 2015). Thus, anxiety and irritability, and the combination of all three AIA symptoms, could potentially be used as non-specific indicators, or ‘gateway symptoms’, to help psychiatrists identify bipolar mania with depressive symptoms (Eberhard and Weiller 2016). At present, the DSM-5 does not highlight or discuss AIA symptoms in the ‘with mixed features’ specifier section. Future iterations of the DSM may want to consider how to discuss the findings of this study and related studies in terms of the mixed specifier. While our findings are not definitive, given the retrospective nature of these analyses and limits of the studies themselves, they do highlight the need for further assessment of the best components to make up the mixed specifier for future DSM-5 editions.

These results support the importance of assessing carefully any patient presenting with significant anxiety, irritability, and agitation for underlying symptoms of depression, especially during mania or hypomanic episodes. While it is routine practice to administer depression scales in some settings, this is not universal. The presence of these symptoms supports that it is a time to review carefully whether they herald less visible symptomology that could inform both clinical care and address patient concerns such as self-harm. As noted in a recent article by Solé and colleagues (2017) and consistent with the findings reported here, indicators of mixity may be key to appropriate treatment choices and addressing suicide prevention effectively (Solé et al. 2017).

Of interest, a recent pilot study examined the use of actigraphy to assess prediction for different bipolar clinical states including mania, depression, and mixed (Scott et al. 2017). In this small sample with 24 h of observation, the researchers were able to predict with 79% accuracy which state a subject was experiencing (Scott et al. 2017). It is to be hoped these types of objective measures may assist us to understand, prevent, and treat mixed symptoms more efficiently.

The presence of anxiety in patients with bipolar disorder has previously been associated with a longer time to remission (Feske et al. 2000). The present analysis extended this observation—showing that patients with anxiety or a combination of all three AIA symptoms are less likely to achieve remission than those without the corresponding symptom(s). Thus, these data support the notion that AIA symptoms identify a more severe or complicated phenotype of mania that is less likely to respond to treatment.

Similarly, patients with three or more depressive symptoms were less likely to achieve remission, although this outcome did not meet the criteria for statistical significance. However, patients with a MADRS Total score of ≥20 showed similar remission rates to those below this depression threshold. The previous post hoc analysis in this same dataset also found that the remission rate (defined as a YMRS Total score of ≤12) was not influenced by the number or severity of depressive symptoms (with the exception of the most severely depressed patients, who showed a *higher* remission rate) (McIntyre et al. 2013a). It may be that those patients who are the most ill may also be the most responsive to the medications under study; and thus most likely to show a larger degree of improvement.

In BD-I (unlike in MDD) there has been relatively little prior investigation into whether patient characteristics (including AIA) within an episode can affect treatment response and other health outcomes (Feske et al. 2000). A recent analysis showed that the presence of AIA symptoms raises the already high suicide risk among patients with BD-I mania with depressive symptoms (Eberhard and Weiller 2016). Additionally, a subtype of bipolar depression, characterised by anxiety and irritability among other symptoms, is associated with a greater likelihood of receiving government financial support, suggesting that this pattern of symptoms is particularly malignant (Perich et al. 2016). From a clinical point of view, given the hazards associated with AIA, clinicians should routinely probe for AIA, and should systematically target these symptoms with treatment. In MDD, for example, many physicians reserve adjunctive antipsychotics for use in patients presenting with agitation or irritability, among other symptoms (McIntyre and Weiller 2015). Furthermore, the 2015 update to the Florida Best Practice Psychotherapeutic Medication Guidelines provides different algorithms for the treatment of MDD according to the presence or absence of mixed features (University of South Florida 2015); this is an unmet need for BD-I.

In the present analysis, both asenapine and olanzapine demonstrated higher remission rates (defined as a YMRS score of <12) than placebo among patients with AIA symptoms. A more stringent definition of remission was also considered (a YMRS score of <8); remission rates were low (<25%) with this definition regardless of treatment, demonstrating the difficulty in reducing symptoms to this level over such a short time period. Asenapine and olanzapine also showed consistent benefits over placebo in terms of mean change from baseline in YMRS Total score among patients with anxiety and all three AIA symptoms. In general, second-generation antipsychotics are efficacious in the treatment of mixed episodes with predominant manic symptoms, and have been described as the treatment of choice in the management of bipolar disorder mixed states (Muralidharan et al. 2013; Fagiolini et al. 2015). In mania with DSM-5 mixed features, asenapine, aripiprazole, and olanzapine have been evaluated and have shown efficacy as potential treatment options (McIntyre et al. 2013a, b; Tohen et al. 2014).

In line with considerations of best treatment choices, a range of guideline efforts for the treatment of bipolar disorder have recently been developed (Stahl et al. 2017; Fountoulakis et al. 2016; Goodwin et al. 2016) or are in revision, such as the CANMAT guidelines (Yatham et al. 2013). The importance of the results reported in this present post hoc analysis is highlighted as the best definition for mixed symptoms is sought and the understanding of the implications for treatment and course of illness is developed. It is clear from both past and more recent efforts that mixed symptoms herald a more severe course of illness, but the optimal definition is still under enquiry (Swann et al. 2013; Barbuti et al. 2017).

This present study is limited in that it is a post hoc analysis of studies that were not designed for this purpose. In particular, as these were regulatory studies, patients with current suicidality or complex comorbidities were excluded from study entry, thus limiting generalizability. In terms of study design, participants were inpatients for at least the first week of treatment, which may have selected for agitation. The results may also have been influenced by the use of benzodiazepines for agitation. Benzodiazepines were not permitted beyond Day 7 of treatment; however, 131 patients (13.6%) in the FAS received one or more doses of benzodiazepines beyond Day 7. The use of proxies for AIA and depressive symptoms was another limitation of the analysis—especially for agitation, which was based on the YMRS increased motor activity–energy item because no agitation item was available. Since irritability and agitation were based upon YMRS items, they were not independent with regard to YMRS remission. It is important to keep in mind that this proxy for depression symptoms (MADRS ≥3), and thus potential mixed symptoms, does not itself assess for the DSM-5 Mixed Specifier criteria and must be viewed as a limit of the study design. Finally, the analysis does not rule out the possibility of pseudospecificity: that AIA and depressive symptoms are discrete, overlapping phenomena.

## Conclusions

Assessment of AIA symptoms in bipolar mania could enable physicians to identify patients with more severe depressive symptoms and who may be at increased risk of suicide, allowing for appropriate intervention. Furthermore, assessment and monitoring of AIA may help physicians to predict which patients may be harder to treat.

## Abbreviations

AIA: anxiety, irritability, and agitation
ANCOVA: analysis of covariance
BD-I: bipolar I disorder
CGI-BP: Clinical Global Impressions-Bipolar version
DSM: Diagnostic and Statistical Manual of Mental Disorders
FAS: full analysis set
MADRS: Montgomery–Åsberg Depression Rating Scale
MDD: major depressive disorder
PANSS: Positive and Negative Syndrome Scale
SD: standard deviation
SE: standard error
YMRS: Young Mania Rating Scale
